# Circulating GPER-1, 8-Iso-Prostaglandin F_2_α, and Raftlin-1 in Hypertensive Women During the Menopausal Transition: Associations with BMI and Diagnostic Performance

**DOI:** 10.3390/jcm15124484

**Published:** 2026-06-10

**Authors:** Ünal Öztürk, Muhammed Mehdi Üremiş, Ergül Belge Kurutaş

**Affiliations:** 1 Department of Cardiology, Medical Faculty, Kahramanmaraş Sütçü İmam University, 46050 Kahramanmaraş, Turkey; 2 Department of Medical Biochemistry, Medical Faculty, Kahramanmaraş Sütçü İmam University, 46050 Kahramanmaraş, Turkey; mmuremis@ksu.edu.tr (M.M.Ü.); ebkurutas@ksu.edu.tr (E.B.K.)

**Keywords:** hypertension, menopausal transition, GPER-1, 8-iso-prostaglandin F_2_α, raftlin-1

## Abstract

**Background:** Hypertension (HTN) is a multifactorial condition involving alterations in vascular signaling, inflammation, and oxidative stress. Women during the menopausal transition may experience increased cardiovascular risk due to hormonal fluctuations, highlighting the need for circulating biomarkers that may assist in identifying HTN-related biological changes. This study aimed to evaluate circulating biomarkers reflecting estrogen signaling, inflammation, and oxidative stress, and to assess their diagnostic accuracy in HTN. **Methods:** This cross-sectional study included 54 treatment-naïve hypertensive women and 30 age-matched normotensive controls, all in the menopausal transition period. Serum levels of GPER-1, 8-iso-PGF_2_α, raftlin-1, and estradiol were measured using ELISA. Associations between biomarkers and body mass index (BMI) were examined using Spearman correlation. Diagnostic discrimination was examined using ROC curve analysis, and optimal cut-off points were determined using the Youden index. **Results:** Serum levels of GPER-1, 8-iso-PGF_2_α, and raftlin-1 were significantly higher in the HTN group compared to controls, while estradiol showed a modest increase. No significant associations were found between BMI and any of the measured biomarkers within the HTN group. ROC analysis demonstrated that 8-iso-PGF_2_α and raftlin-1 showed the highest diagnostic accuracy for distinguishing HTN patients from controls, followed by GPER-1, whereas estradiol showed limited diagnostic value. High sensitivity and specificity values further supported the diagnostic potential of 8-iso-PGF_2_α and raftlin-1. **Conclusions:** These findings suggest that serum raftlin-1 and 8-iso-PGF_2_α levels may serve as potential biomarkers for distinguishing hypertension in women during the menopausal transition, while GPER-1 also demonstrated good diagnostic accuracy. The lack of association with BMI suggests relative independence from adiposity-related effects. However, further validation in larger and well-characterized cohorts is required.

## 1. Introduction

Hypertension (HTN) is one of the most prevalent cardiovascular risk factors worldwide and is associated with vascular remodeling, endothelial dysfunction, and progressive organ damage [1]. Alterations in signaling pathways that regulate vascular tone [2], chronic low-grade inflammation [3], and increased oxidative stress [4] are central to HTN pathophysiology, and these processes are often reflected by changes in circulating biomarker levels. Although the diagnosis of HTN primarily relies on office, home, or ambulatory blood pressure measurements, blood pressure values alone may not adequately reflect the underlying biological heterogeneity, vascular injury, or early pathophysiological alterations associated with disease progression. Therefore, there is growing interest in identifying circulating biomarkers that may complement conventional diagnostic approaches by providing additional information regarding disease mechanisms, cardiovascular risk, and potential prognosis. This may be particularly relevant in women during the menopausal transition, a period characterized by marked hormonal fluctuations and increasing cardiovascular vulnerability.

Research on estrogen signaling and the modulatory effects of estrogen receptors on the cardiovascular system has intensified in recent years. The G protein-coupled estrogen receptor-1 (GPER-1) mediates rapid, non-genomic estrogen signaling and may influence vascular tone, inflammatory responses, and oxidative stress, suggesting a potential role in the pathogenesis of hypertension [5]. In women, the menopausal transition represents a critical period characterized by fluctuating and progressively declining estrogen levels, which may contribute to increased cardiovascular risk and alterations in vascular homeostasis [6]. Given that estrogen deficiency is associated with increased cardiovascular risk in postmenopausal women [7], investigation of GPER-1 could yield important insights into sex-specific mechanisms of HTN, particularly during this transitional phase.

Oxidative stress markers remain important functional indicators in cardiovascular research. 8-iso-prostaglandin F_2_α (8-iso-PGF_2_α), a stable product of lipid peroxidation, reliably reflects systemic oxidative stress and has demonstrated diagnostic and prognostic relevance in cardiovascular disease [8]. Raftlin-1, a protein localized to membrane lipid rafts, modulates Toll-like receptor-mediated signaling and innate immune responses and has been implicated in inflammatory and proangiogenic pathways relevant to vascular biology [9]. These properties render raftlin-1 a candidate biomarker for vascular inflammation and hypertension-related pathophysiology.

Direct, head-to-head comparisons of biomarkers reflecting distinct pathophysiological mechanisms (estrogen signaling, oxidative stress, and inflammation) in women with hypertension during the menopausal transition remain limited. In addition, it is still unclear which biomarkers may provide the greatest diagnostic relevance in this hormonally dynamic population. Body mass index (BMI), an important marker of adiposity [10], can influence estrogen activity, inflammatory tone, and oxidative stress and therefore may modify the associations between these biomarkers and hypertension. However, the relationships between BMI and these circulating biomarkers have not been sufficiently characterized in women during the menopausal transition. Addressing these gaps may improve understanding of the biological heterogeneity of HTN and help identify biomarkers with potential clinical relevance for risk assessment and disease characterization in women at increased cardiovascular risk. Therefore, the present study aimed to compare serum levels of GPER-1, 8-iso-PGF_2_α, raftlin-1, and estradiol between hypertensive and normotensive women during the menopausal transition, to evaluate the relationships between these biomarkers and BMI, and to assess their diagnostic accuracy for hypertension using ROC curve analysis.

## 2. Materials and Methods

### 2.1. Study Design and Participants

This cross-sectional study was conducted at the Cardiology Outpatient Clinic of Kahramanmaraş Sütçü İmam University Research Hospital. A total of 54 HTN patients and 30 normotensive controls were included. Women aged 40–60 years who were in the menopausal transition period were eligible for inclusion. The menopausal transition was defined based on age (40–60 years) and clinical menstrual history, including irregular menstrual cycles, in accordance with established clinical criteria. However, hormonal confirmation using follicle-stimulating hormone (FSH), luteinizing hormone (LH), or estradiol-based staging was not performed, and therefore classification was based on clinical assessment rather than biochemical confirmation. Among these, patients who had been newly diagnosed with hypertension were consecutively enrolled.

Blood pressure measurements, including systolic and diastolic blood pressure, were used for the diagnosis of hypertension. Participants were classified as hypertensive if systolic blood pressure was ≥130 mmHg and/or diastolic blood pressure was ≥80 mmHg, based on the average of at least two office measurements obtained after a minimum 5 min rest using a validated automated device, in line with current international recommendations [11]. Only treatment-naïve patients who had not previously received antihypertensive therapy were included.

A normotensive control group consisting of age-matched women in the menopausal transition period was also recruited. Normotension was defined as systolic blood pressure <130 mmHg and diastolic blood pressure <80 mmHg, assessed using the same standardized blood pressure measurement protocol as applied to the HTN group.

### 2.2. Blood Sample Collection and Processing

After obtaining written informed consent, fasting venous blood samples were collected from all participants in the morning. Venous blood was collected into serum separator tubes, followed by centrifugation at 4000 rpm for 10 min. The resulting serum was then aliquoted and stored at −80 °C until further analysis.

### 2.3. Biochemical Analyses

Serum levels of GPER-1, 8-iso-PGF_2_α, raftlin-1, and estradiol were quantified using commercially available enzyme-linked immunosorbent assay (ELISA) kits (MyBioSource, San Diego, CA, USA) following the manufacturer’s protocols. All assays were performed in duplicate, and optical density was measured with a microplate reader. Concentrations were determined from analyte-specific standard curves.

### 2.4. Anthropometric Measurements

Body weight (kg) and height (m) were measured using standard procedures, and body mass index (BMI) was calculated as weight divided by height squared (kg/m^2^). BMI was included as an anthropometric parameter to examine its association with circulating biomarker levels and its potential relationship with hypertension status.

### 2.5. Statistical Analysis

Statistical analyses were performed using GraphPad Prism 10 software. The normality of data distribution for continuous variables was assessed using the Shapiro–Wilk test. Normally distributed variables were expressed as mean ± standard deviation and compared between groups using Welch’s *t*-test. Non-normally distributed variables were presented as mean ± standard deviation and compared using the Mann–Whitney U test. Categorical variables were expressed as number (percentage) and analyzed using Fisher’s exact test.

Associations between BMI and serum biomarker levels within the HTN group were assessed using Spearman rank correlation analysis. The diagnostic accuracy of GPER-1, estradiol, raftlin-1, and 8-iso-PGF_2_α for distinguishing HTN patients from normotensive controls was evaluated using receiver operating characteristic (ROC) curve analysis. A *p*-value < 0.05 was considered statistically significant.

### 2.6. Ethical Approval

The study protocol was approved by the Institutional Ethics Committee of Kahramanmaraş Sütçü İmam University (2024/34-09), and the study was conducted in accordance with the principles of the Declaration of Helsinki. Written informed consent was obtained from all participants prior to inclusion.

## 3. Results

Demographic and clinical characteristics of the study population are presented in Table 1. Age distribution was comparable between the control and HTN groups (*p* = 0.12). BMI, systolic blood pressure (SBP), and diastolic blood pressure (DBP) values were significantly higher in HTN patients compared with normotensive controls (all *p* < 0.001). No significant differences were observed between the groups regarding diabetes mellitus status, hyperlipidemia status, fasting glucose levels, or lipid profile parameters (all *p* > 0.05).

### 3.1. Clinical and Biochemical Characteristics of the Study Groups

**Figure 1:**
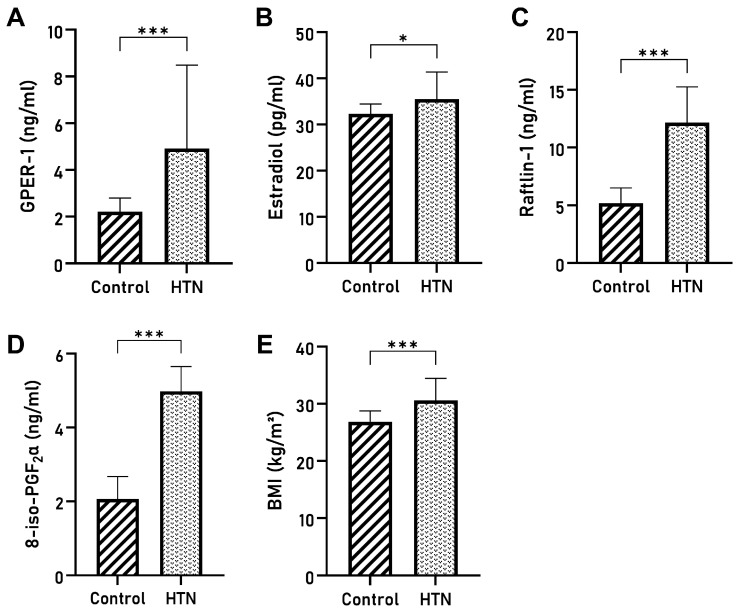
Comparison of Serum Biomarker Levels and BMI Between HTN and Control Groups. Serum levels of (**A**) GPER-1, (**B**) estradiol, (**C**) raftlin-1, (**D**) 8-iso-prostaglandin F_2_α (8-iso-PGF_2_α), and (**E**) body mass index (BMI) were compared between hypertensive (HTN) patients and normotensive controls. Data are presented as mean ± standard deviation. Statistical comparisons were performed using appropriate parametric or non-parametric tests. * *p* < 0.05, *** *p* < 0.001 versus control group.

Serum levels of GPER-1, 8-iso-PGF_2_α, raftlin-1, and estradiol as well as BMI, were compared between HTN patients and normotensive control subjects (Figure 1). HTN patients exhibited significantly higher serum GPER-1 concentrations compared with the control group (*p* < 0.001). Serum 8-iso-PGF_2_α and raftlin-1 levels were also markedly elevated in the HTN group (both *p* < 0.001). Estradiol levels showed a modest but statistically significant increase in HTN patients compared with controls (*p* < 0.05).

### 3.2. Correlation Analysis Between BMI and Biomarkers in HTN Patients

Spearman rank correlation analysis was performed to evaluate the relationships between BMI and circulating biomarker levels within the HTN group. No significant correlations were observed between BMI and serum GPER-1 (r = 0.048, *p* = 0.728), estradiol (r = −0.150, *p* = 0.278), raftlin-1 (r = 0.009, *p* = 0.948), or 8-iso-PGF_2_α (r = 0.110, *p* = 0.428).

**Figure 2:**
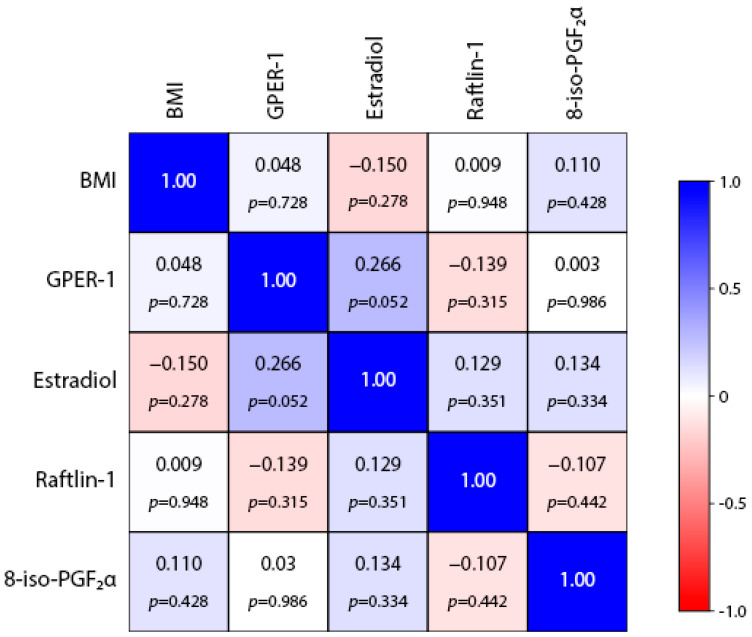
Correlation Matrix of BMI and Circulating Biomarkers in the HTN Group. Heatmap representing pairwise Spearman correlation coefficients among BMI, GPER-1, estradiol, raftlin-1, and 8-iso-PGF_2_α in the hypertensive (HTN) group. Color intensity indicates the strength and direction of correlations (blue: positive, red: negative). Numerical values within each cell represent Spearman correlation coefficients (r), and the corresponding *p*-values are also displayed. Spearman rank correlation analysis was used to assess associations between variables.

These findings were further visualized using a correlation heatmap illustrating pairwise Spearman correlation coefficients among BMI, GPER-1, estradiol, raftlin-1, and 8-iso-PGF_2_α (Figure 2). The heatmap demonstrated weak and non-significant correlations between BMI and all evaluated biomarkers, as well as generally low inter-marker correlations, suggesting that these biomarkers may reflect partially distinct biological processes in HTN patients.

### 3.3. Diagnostic Performance of Biomarkers for Hypertension

**Figure 3:**
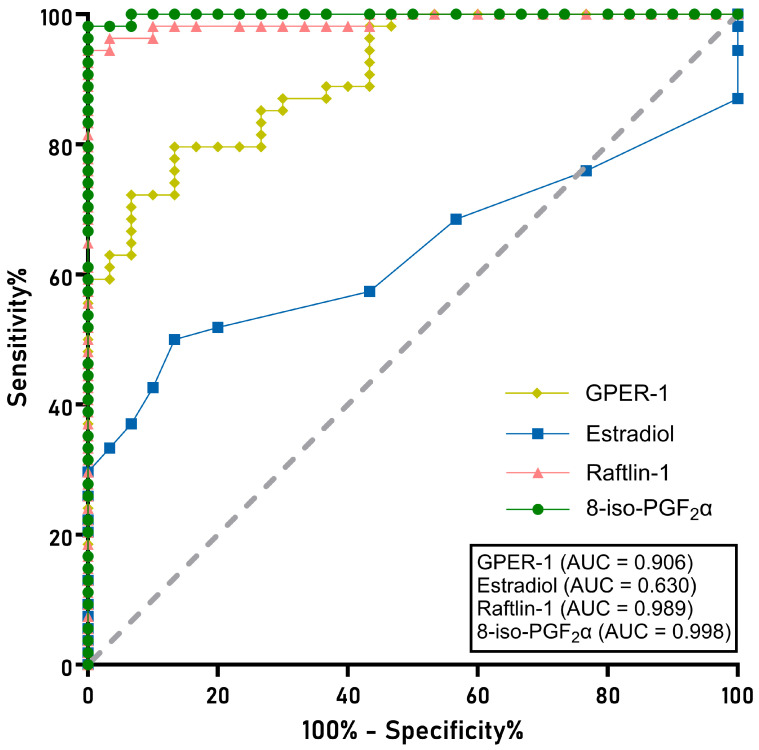
Receiver Operating Characteristic (ROC) Curve Analysis of Biomarkers for Hypertension. Receiver operating characteristic (ROC) curves comparing the diagnostic accuracy of GPER-1, estradiol, raftlin-1, and 8-iso-prostaglandin F_2_α (8-iso-PGF_2_α) for distinguishing hypertensive (HTN) patients from normotensive controls. The area under the curve (AUC) values with 95% confidence intervals were as follows: GPER-1, 0.906 (95% CI: 0.8467–0.9669); estradiol, 0.630 (95% CI: 0.5145–0.7472); raftlin-1, 0.989 (95% CI: 0.9723–1.000); and 8-iso-PGF_2_α, 0.998 (95% CI: 0.9954–1.000). The dashed diagonal line represents the line of no discrimination. Detailed AUC values, optimal cut-off values, sensitivity, and specificity are provided in Table 2.

Receiver operating characteristic (ROC) curve analysis was conducted to assess the diagnostic performance of serum GPER-1, estradiol, raftlin-1, and 8-iso-PGF_2_α for distinguishing HTN patients from normotensive controls (Figure 3). GPER-1 showed good diagnostic value (AUC = 0.906), whereas estradiol demonstrated limited diagnostic value (AUC = 0.630). Raftlin-1 (AUC = 0.989) and 8-iso-PGF_2_α (AUC = 0.998) exhibited excellent diagnostic accuracy for distinguishing HTN from normotensive individuals. Detailed optimal cut-off values, sensitivity, and specificity are presented in Table 2.

Overall, these findings indicate that while estradiol alone has limited diagnostic utility, serum GPER-1, raftlin-1, and particularly 8-iso-PGF_2_α demonstrated strong diagnostic accuracy for distinguishing HTN patients from normotensive individuals. These biomarkers may have potential utility for biomarker-based characterization of hypertension in women during the menopausal transition; however, further validation in larger cohorts is required.

## 4. Discussion

In this study, serum levels of GPER-1, 8-iso-PGF_2_α, and raftlin-1 were found to be significantly elevated in HTN women during the menopausal transition compared to normotensive controls, and these biomarkers appeared to be largely independent of adiposity- and metabolism-related parameters. In addition, raftlin-1 and 8-iso-PGF_2_α demonstrated high diagnostic performance in distinguishing HTN. These findings suggest that endocrine, inflammatory, and oxidative stress pathways contribute to the pathophysiology of HTN through interconnected yet partially distinct biological mechanisms.

The observation of significantly higher serum GPER-1 levels in HTN women highlights the potential role of estrogen-related signaling in this context. GPER-1 activation contributes to vasodilation and reduced peripheral resistance through mechanisms including cAMP production, calcium mobilization, and stimulation of endothelial nitric oxide synthase [12,13]. Consistent with these protective effects, previous studies have reported lower serum GPER-1 levels in HTN postmenopausal women and identified GPER-1 as an independent protective factor in multivariate analyses [14]. However, GPER-1 has also been shown to exert vasoconstrictive and sodium-retentive effects in the presence of aldosterone [15]. The elevated GPER-1 levels observed in our study may reflect a context-specific aspect of GPER-1 biology during the menopausal transition. Fluctuating and gradually declining estrogen levels in perimenopause may induce compensatory upregulation of GPER-1 expression in vascular and immune cells [16]. In addition, increased shedding of membrane-bound receptors under conditions of chronic inflammation and oxidative stress may contribute to higher circulating GPER-1 levels, as supported by the concurrent elevation of raftlin-1 and 8-iso-PGF_2_α in our cohort. However, elevated serum levels do not necessarily indicate preserved receptor functionality. Therefore, circulating GPER-1 may reflect compensatory expression or receptor shedding rather than functional signaling capacity. Such dissociation between receptor abundance and function has also been described for other membrane receptors under inflammatory conditions [17].

The elevated raftlin-1 levels observed in the HTN group suggest activation of immune-inflammatory pathways. Raftlin-1 is an adaptor protein localized in lipid raft microdomains and plays a critical role in innate immune signaling [18]. Initial clinical evidence for its role as a systemic inflammatory marker was provided by Lee et al., who reported markedly increased circulating raftlin-1 levels in sepsis, associated with endothelial dysfunction [19]. In the cardiovascular context, lipid raft integrity is essential for organizing NADPH oxidase subunits in endothelial membranes, and disruption of these structures promotes oxidative stress and impairs endothelium-dependent vasodilation [20]. Thus, increased raftlin-1 levels in our cohort may reflect lipid raft-mediated inflammatory activation contributing to endothelial dysfunction. A study in severe COVID-19 patients reported concurrent elevations of raftlin-1 and 8-iso-PGF_2_α, linking these biomarkers within a shared framework of immune activation and oxidative lipid damage [21]. The parallel increase in these biomarkers in our study supports the possibility of a bidirectional interaction between inflammation and oxidative stress.

8-iso-PGF_2_α is generated through free radical-driven, non-enzymatic peroxidation of arachidonic acid and is widely regarded as a dependable indicator of systemic oxidative stress in cardiovascular conditions [22]. In addition to its role as a biomarker, it is a biologically active mediator that can directly induce vasoconstriction, platelet activation, and vascular smooth muscle proliferation [23]. Elevated levels of 8-iso-PGF_2_α in HTN individuals have been consistently reported in previous studies [23,24], and its plasma levels have been associated with endothelial dysfunction in resistant HTN [25]. Our findings are consistent with increased oxidative stress during the menopausal transition, likely related to the decline in estrogen-mediated antioxidant defenses. The simultaneous elevation of raftlin-1 and 8-iso-PGF_2_α may indicate a self-amplifying cycle, in which lipid raft-mediated Toll-like receptor signaling enhances NADPH oxidase activity and reactive oxygen species production, thereby promoting lipid peroxidation and further increasing 8-iso-PGF_2_α levels, ultimately exacerbating endothelial dysfunction [26].

Although BMI was higher in HTN patients compared to normotensive controls, consistent with the known contribution of adiposity to elevated blood pressure, no significant correlations were observed between BMI and any of the investigated biomarkers within the HTN group. In addition, diabetes mellitus status, hyperlipidemia status, fasting glucose levels, and lipid profile parameters did not differ significantly between the groups. These findings suggest that the observed elevations in GPER-1, raftlin-1, and 8-iso-PGF_2_α may not be solely explained by adiposity- or metabolism-related alterations, although residual metabolic confounding cannot be completely excluded.

ROC analyses, with threshold values selected based on the highest Youden index, showed that 8-iso-PGF_2_α (AUC = 0.998) and raftlin-1 (AUC = 0.989) had the strongest diagnostic performance, followed by GPER-1 (AUC = 0.906), whereas estradiol demonstrated limited diagnostic value (AUC = 0.630). The high sensitivity and specificity values observed for 8-iso-PGF_2_α and raftlin-1 further support their ability to distinguish HTN from normotensive individuals. This hierarchical pattern suggests that inflammatory and oxidative stress-related markers may provide stronger diagnostic performance than hormonal markers in this context. In addition, estradiol findings should be interpreted cautiously because circulating estrogen levels may be influenced by hormonal stage, adiposity, and metabolic factors that were not comprehensively controlled in the present study. Although several metabolic parameters were additionally evaluated in the present study, more comprehensive metabolic and endocrine profiling may further clarify the potential influence of metabolic status on circulating biomarker levels. However, these findings should be validated in larger and independent cohorts before clinical application. These biomarkers, particularly 8-iso-PGF_2_α and raftlin-1, may have potential utility individually or in combination for early identification of HTN and cardiovascular risk stratification in perimenopausal women.

The rationale for incorporating circulating biomarkers in this setting stems from the limitations of blood pressure-based diagnosis. Office blood pressure measurements show considerable variability and may be influenced by white-coat and masked hypertension, potentially leading to misclassification when diagnosis relies solely on threshold values [11,27]. Moreover, blood pressure reflects the hemodynamic manifestation of the disease rather than the underlying vascular injury, oxidative burden, and inflammatory activation; consequently, individuals with similar blood pressure values may differ substantially in their degree of endothelial dysfunction. Serum biomarkers reflecting distinct pathophysiological axes, namely estrogen signaling (GPER-1), oxidative stress (8-iso-PGF_2_α), and lipid raft-mediated inflammation (raftlin-1), may therefore provide biological information that is not captured by blood pressure measurements alone. The high diagnostic accuracy of 8-iso-PGF_2_α and raftlin-1 observed in our cohort supports this complementary value. This may be particularly relevant during the menopausal transition, when declining estrogen levels are associated with accelerated vascular aging and enhanced oxidative and inflammatory signaling [6,28].

Beyond diagnosis, these findings may have implications for cardiovascular risk stratification during the menopausal transition, a period characterized by increasing cardiovascular vulnerability that may warrant earlier and more individualized risk assessment [28]. Oxidative stress and vascular inflammation are key contributors to endothelial dysfunction and cardiovascular disease progression, and 8-iso-PGF_2_α has previously been associated with both predicted 10-year cardiovascular risk and endothelial dysfunction in hypertensive populations [23,25]. Within this context, the marked elevations of 8-iso-PGF_2_α and raftlin-1 may reflect a greater burden of vascular injury and increased cardiovascular vulnerability. As these biomarkers appeared to be largely independent of BMI and routine lipid parameters in our cohort, they may provide complementary information beyond conventional clinical variables. A multimarker approach integrating oxidative stress and inflammation-related biomarkers may therefore help identify biologically higher-risk phenotypes among women with hypertension during the menopausal transition. However, prospective studies linking these biomarkers to incident cardiovascular events are required before their prognostic utility can be established.

Several limitations of this study should be acknowledged. The cross-sectional design does not allow conclusions regarding temporal relationships or causality between biomarker changes and blood pressure elevation. Although the sample size was sufficient for primary comparisons, the overall sample size was relatively small and the groups were unevenly distributed, which may have introduced heterogeneity and limited the statistical power of subgroup analyses. Therefore, the findings should be interpreted with caution and validated in larger, more balanced cohorts. In addition, biomarker measurements were obtained at a single time point, which may not fully capture biological variability. Menopausal status was determined based on age range and clinical menstrual history rather than biochemical confirmation using hormonal markers such as follicle-stimulating hormone (FSH) or luteinizing hormone (LH) levels. Therefore, some degree of overlap or misclassification between perimenopausal and early postmenopausal stages cannot be excluded, which may have contributed to biological heterogeneity within the study population. Although metabolic and lipid-related variables were evaluated, residual confounding associated with unmeasured metabolic or endocrine factors cannot be completely excluded. Future studies with larger, well-characterized cohorts and longitudinal designs are needed to validate the proposed diagnostic cut-off values and to assess the predictive value of these biomarkers for incident cardiovascular outcomes.

In conclusion, serum levels of GPER-1, 8-iso-PGF_2_α, and raftlin-1 were elevated in HTN women during the menopausal transition and may reflect distinct pathophysiological pathways associated with hypertension. The observed diagnostic performance of raftlin-1 and 8-iso-PGF_2_α suggests a possible contribution of lipid raft-mediated inflammation and oxidative stress in this condition. Elevated GPER-1 levels may reflect compensatory upregulation or receptor shedding in the setting of altered estrogenic signaling, although further mechanistic studies are needed. The lack of association with BMI suggests that these processes may be relatively independent of adiposity and routinely assessed metabolic parameters. By reflecting oxidative and inflammatory vascular alterations that may precede sustained blood pressure elevation, 8-iso-PGF_2_α and raftlin-1 could aid the early detection of hypertension and help identify perimenopausal women at greater cardiovascular risk. A multimarker strategy may thus complement conventional blood pressure-based evaluation for earlier risk stratification in this vulnerable period. Overall, these findings indicate that multibiomarker approaches integrating inflammatory and oxidative stress-related pathways may have potential for improving the understanding and identification of hypertension in perimenopausal women. However, larger and longitudinal studies are required before clinical applicability can be established.

## Figures and Tables

**Table 1:** Demographic and clinical characteristics of the control and hypertensive (HTN) groups.

Parameter	Control	HTN	*p*
Age (years)	43.9 ± 6.98	46.2 ± 5.98	0.12
BMI (kg/m^2^)	26.8 ± 1.93	30.6 ± 3.83	<0.001
SBP (mmHg)	124 ± 5.21	137 ± 7.03	<0.001
DBP (mmHg)	76.4 ± 4.01	84.4 ± 6.25	<0.001
DM, *n* (%)	6 (20%)	10 (18.52%)	>0.99
Hyperlipidemia, *n* (%)	6 (20%)	14 (25.93%)	0.29
Fasting glucose (mg/dL)	103 ± 27.5	108 ± 27.9	0.24
Total cholesterol (mg/dL)	172 ± 42.2	177 ± 42.6	0.60
TG (mg/dL)	149 ± 38.0	144 ± 91.9	0.73
HDL-C (mg/dL)	42.4 ± 7.93	39.7 ± 9.82	0.20
LDL-C (mg/dL)	107 ± 27.7	110 ± 29.8	0.65

Data are presented as mean ± standard deviation or number (percentage). Comparisons between groups were performed using Welch’s *t*-test or Mann–Whitney U test for continuous variables, depending on data distribution, and Fisher’s exact test for categorical variables. Abbreviations: BMI, body mass index; SBP, systolic blood pressure; DBP, diastolic blood pressure; DM, diabetes mellitus; TG, triglyceride; HDL-C, high-density lipoprotein cholesterol; LDL-C, low-density lipoprotein cholesterol; HTN, hypertension.

**Table 2:** Diagnostic performance of serum biomarkers for distinguishing hypertensive patients from normotensive controls.

Biomarker	AUC	Cut-Off	Sensitivity	Specificity
GPER-1	0.906	2.94 ng/mL	72%	93%
Estradiol	0.630	35.5 pg/mL	43%	90%
Raftlin-1	0.989	7.015 ng/mL	96%	97%
8-iso-PGF_2_α	0.998	3.56 ng/mL	98%	100%

Values include area under the curve (AUC), optimal cut-off values determined by the Youden index, sensitivity, and specificity for each biomarker. Receiver operating characteristic (ROC) curve analysis was used to evaluate diagnostic performance. Abbreviation: AUC, area under the curve.

## Data Availability

The data generated and/or analyzed during this study can be obtained from the corresponding author upon reasonable request.
